# PI3K/AKT/mTOR inhibition in combination with doxorubicin is an effective therapy for leiomyosarcoma

**DOI:** 10.1186/s12967-016-0814-z

**Published:** 2016-03-08

**Authors:** Yael Babichev, Leah Kabaroff, Alessandro Datti, David Uehling, Methvin Isaac, Rima Al-awar, Michael Prakesch, Ren X. Sun, Paul C. Boutros, Rosemarie Venier, Brendan C. Dickson, Rebecca A. Gladdy

**Affiliations:** Lunenfeld-Tanenbaum Research Institute, Mount Sinai Hospital, Toronto, M5G 1X5 Canada; Sinai-McLaughlin Assay and Robotic Technologies Facility, Lunenfeld-Tanenbaum Research Institute, Toronto, M5G 1X5 Canada; Department of Agricultural, Food, and Environmental Sciences, University of Perugia, 06121 Perugia, Italy; Drug Discovery Group, Ontario Institute for Cancer Research, Toronto, M5G 0A3 Canada; Department of Pharmacology and Toxicology, University of Toronto, Toronto, M5S 1A8 Canada; Informatics and Biocomputing Program, Ontario Institute for Cancer Research, Toronto, M5G 0A3 ON Canada; Department of Medical Biophysics, University of Toronto, Toronto, M5S 1A1 ON Canada; Department of Pathology and Laboratory Medicine, Mount Sinai Hospital, Toronto, M5G 1X5 ON Canada; Department of Surgery, University of Toronto, Toronto, M5S 1A1 Canada; Institute of Medical Science, University of Toronto, Toronto, M5S 1A1 Canada; Cancer Stem Cell Program, Ontario Institute for Cancer Research, Toronto, M5G 0A3 ON Canada; Lunenfeld-Tanenbaum Research Institute, 25 Orde Street, Room 5-1015-2, Toronto, ON M5T 3H7 Canada

**Keywords:** Leiomyosarcoma, PI3K, mTOR, Drug discovery, Sarcoma, Doxorubicin

## Abstract

**Background:**

Leiomyosarcoma (LMS) is a common type of soft tissue sarcoma that responds poorly to standard chemotherapy. Thus the goal of this study was to identify novel selective therapies that may be effective in leiomyosarcoma by screening cell lines with a small molecule library comprised of 480 kinase inhibitors to functionally determine which signalling pathways may be critical for LMS growth.

**Methods:**

LMS cell lines were screened with the OICR kinase library and a cell viability assay was used to identify potentially effective compounds. The top 10 % of hits underwent secondary validation to determine their EC_50_ and immunoblots were performed to confirm selective drug action. The efficacy of combination drug therapy with doxorubicin (Dox) in vitro was analyzed using the Calcusyn program after treatment with one of three dosing schedules: concurrent treatment, initial treatment with a selective compound followed by Dox, or initial treatment with Dox followed by the selective compound. Single and combination drug therapy were then validated in vivo using LMS xenografts.

**Results:**

Compounds that targeted PI3K/AKT/mTOR pathways (52 %) were most effective. EC_50_s were determined to validate these initial hits, and of the 11 confirmed hits, 10 targeted PI3K and/or mTOR pathways with EC_50_ values <1 μM. We therefore examined if BEZ235 and BKM120, two selective compounds in these pathways, would inhibit leiomyosarcoma growth in vitro. Immunoblots confirmed on-target effects of these compounds in the PI3K and/or mTOR pathways. We next investigated if there was synergy with these agents and first line chemotherapy doxorubicin (Dox), which would allow for earlier introduction into patient care. Only combined treatment of BEZ235 and Dox was synergistic in vitro. To validate these findings in pre-clinical models, leiomyosarcoma xenografts were treated with single agent and combination therapy. BEZ235 treated xenografts (n = 8) demonstrated a decrease in tumor volume of 42 % whereas combining BEZ235 with Dox (n = 8) decreased tumor volume 68 % compared to vehicle alone.

**Conclusions:**

In summary, this study supports further investigation into the use of PI3K and mTOR inhibitors alone and in combination with standard treatment in leiomyosarcoma patients.

**Electronic supplementary material:**

The online version of this article (doi:10.1186/s12967-016-0814-z) contains supplementary material, which is available to authorized users.

## Background

Sarcomas are a diverse group of malignant mesenchymal neoplasms with over 50 histologically distinct subtypes [1]. They can be broadly classified in two groups: those containing simple karyotypic defects including recurrent translocations or those with complex cytogenetic lesions characterised by multiple altered genes, such as leiomyosarcoma (LMS) [1, 2]. LMS accounts for 11 % of soft tissue sarcomas (STS) and although its genetic basis is not fully characterized, common genetic abnormalities include loss of function mutations in p53 or PTEN and activating mutations in the PI3K/AKT/mTOR pathways [3–6]. Conventional treatment of LMS often involves surgical resection, chemotherapy and/or radiation [2]. Unfortunately, current 5-year disease specific survival for resectable, non-metastatic LMS is 60 % for retroperitoneal and 75 % in extremity patients [7, 8]. Since the main patterns of failure are metastatic disease and multifocal local recurrence, effective chemotherapeutic options are essential to improve more durable disease control.

First-line LMS chemotherapy currently consists of doxorubicin (Dox), an anthracycline that inhibits topoisomerase II thereby disrupting DNA repair, in combination with ifosfamide [9]. However, locally advanced, recurrent or metastatic uterine LMS shows only a 25 % response rate to Dox when administered at a dose of 60–80 mg/m^2^ IV every 3 weeks [10]. The use of Dox in cancer therapy is limited by cardiotoxicity, leucopenia, thrombocytopenia and the development of resistance [10]. Another commonly used regimen is gemcitabine (900 mg/m^2^ day 1 and 8) and docetaxel (100 mg/m^2^ day 8), which has response rates ranging from 27 to 53 % in uterine and non-uterine leiomyosarcoma [11]. Therefore, overall poor response rates and significant toxicity in LMS patients implores that more effective, less toxic selective therapies be developed to improve patient outcomes.

In an effort to increase efficacy of treatment, selective inhibitors are being widely developed to target tumor-specific molecular pathways. Recently, STS and other solid tumors including breast, lung and colon cancer have been characterized as exhibiting PI3K/AKT/mTOR dysregulation [12–16]. The PI3K/AKT/mTOR pathways are activated by receptor tyrosine kinases (RTK) that transmit extracellular signals from the tumor microenvironment. These pathways can be dysregulated not only by hyperactivation of growth factor signalling but also through activating or loss of function mutations affecting key molecules such as AKT and PTEN. Targeting these pathways has become a rapidly expanding field in drug development as several small molecule inhibitors targeting these proteins have recently been studied in clinical trials (BKM120, BEZ235 and MK2206) [17–20]. However, complex regulation of the PI3K/mTOR pathways includes feedback loops allowing targeted proteins to be circumvented. Mounting evidence of cross-talk/redundant functions between pathways MAPK and ERK have been linked to the development of adaptive resistance when targeting the PI3K/mTOR pathways [21, 22]. Thus, it has been suggested that small molecules targeting multiple pathways or combining these agents with conventional cytotoxic agents may thwart the development of resistance and result in more durable drug therapy [23].

To identify novel selective therapies for LMS that are effective and potentially less toxic, we utilized a selective drug library of 480 small molecule kinase inhibitors. Based on our initial screen with validation studies in vitro, we report the novel finding that PI3K/AKT/mTOR pathways are functionally important for LMS cell survival. Furthermore, we discovered that BEZ235, a dual PI3K/mTOR inhibitor, significantly inhibited LMS growth in vivo. Finally, this small molecule is synergistic with the current standard of care, doxorubicin, suggesting a promising combination therapy for LMS patients.

## Methods

### Cell line culture conditions and characterisation

Following Research Ethics Board consent at Mount Sinai Hospital (Creation of a Sarcoma Cell Line Biorepository from Human Tissue MSH REB# 10-0310-C), the LMS cell line, STS39, was derived by manual dissociation of a patient sample (pelvic LMS) which was incubated overnight at 37 °C in supplemented DMEM/F-12 10 % FBS (Life Technologies, Burlington, CA) with 2 μg/ml collagenase (SigmaAldrich, Oakville, CA). Cells were repeatedly aspirated to create single cell suspension, centrifuged at 1000 rpm for 5 min, and plated to a T75 flask. SKLMS1 cells obtained from ATCC were cultured in DMEM (Life Technologies), 10 % FBS media. Cells were serially passaged with standard conditions and analysed for copy number variation using array comparative genomic hybridization (aCGH) on the Genome-Wide Human SNP Array 6.0, and mapped using short tandem repeat (STR) analysis (The Centre for Applied Genomics (TCAG) Sick Kids, Toronto, CA). Immunocytochemistry was performed by staining for desmin [clone DE-U-10] (AbCam, Toronto ON, CAN) 1:200, and smooth muscle actin (SMA) (clone 1A4) (Dako, Burlington, CA) 1:200, mouse IgG (Santa Cruz Biotechnology, Dallas, US) 1:400 was used to control for background. To ensure the cell line was representative of the tumor of origin, immunocytochemistry of STS39 cells was matched to patient tumor immunohistochemistry performed by the Department of Pathology at Mount Sinai Hospital. Ki67 and TUNEL staining were quantitated by ImageJ (148v), while p-AKTS473 staining was assessed by staining distribution and intensity. Sequencing of exon 9 and 20 in the p85 subunit of PI3K (Ensembl:ENSG00000145675), and exon 14 and 15 of mTOR (Ensembl:ENSG00000198793) was performed by TCAG.

### Kinase library

A 480 compound kinase library was assembled by the drug discovery group at the Ontario Institute for Cancer Research (OICR). All drugs were used as 100 % solutions in DMSO. The drugs encompassed a variety of targets including but not limited to PI3K/AKT, EGFR/ErbB2, CDKs, and GSK3. BEZ235 and BKM120 were supplied by the Medicinal Chemistry Platform or purchased from ChemieTek (Indianapolis, US).

### Primary screen

A primary drug screen was performed at the SMART Facility in the Lunenfeld–Tanenbaum Research Institute (LTRI) by seeding SKLMS1 and STS39 cells at a density of 600 cells/well and 700 cells/well respectively, in 384 well plates (VWR). Plates were incubated for 6 h to ensure cell attachment, at which time drugs were pinned at 3 concentrations, 0.2, 1 and 5. Following incubation at 37 °C for 96 h, 45 μl of AlamarBlue^®^ (LifeTechnologies) was added. The fluorescence intensity was then measured after 5 h on a BMG Pherastar plate reader. Cells grown in 0.1 % dimethylsulfoxide (DMSO) (SigmaAldrich) alone served as a control to which the results were normalized, while media with no cells was used for background correction.

### Drug selection criteria and hit validation

Hits from the primary screen were defined as the top 10 % of drugs that caused a signal decrease as compared to controls across all three dose groups in both cell lines. The top 10 % of hits were validated by performing a 10-point, threefold serial dilution EC_50_ curve. Stock compounds in the form of lyophilized powder were reconstituted with DMSO to a concentration of 50 mM. SKLMS1 and STS39 cells were seeded at a density of 5000 c/well and 10,000 c/well respectively, into 96 well plates (Fisher, Ottawa, CA) and compounds were added at final concentrations ranging from 0.25 nM to 5 μM and incubated for 72 h. An ATPlite assay (PerkinElmer) was then performed according to manufacturer’s description and fluorescence read on a PerkinElmer Enspire 2300 multimode plate reader. EC_50_ curves were generated and analysed using GraphPad Prism 4.0.

### Immunoblots

SKLMS1 and STS39 cells were seeded in six well plates at a density of 250,000 c/well and 500,000 c/well respectively. Cells were treated with BKM120 or BEZ235 (5–1000 nM) for 72 h prior to harvest. Following a cold PBS (Sigma) rinse, cells were lysed for 20 min on ice with RIPA buffer (50 mM pH 7.4 Tris–HCl, 150 mM NaCl, 1 % NP-40, 1 mM EDTA) supplemented with phosphatase and protease inhibitors (Sigma). Protein concentration was measured with DC Protein concentration assay (BioRad). Electrophoresis was performed using MiniProtean TGX gels (Bio-Rad) and transferred to PVDF by wet transfer. Immunoblots were performed with the following antibodies: p-AKT^S473^, total AKT, p-S6K^T389^, total S6K, p-4EBP1^T37/46^, total 4EBP1, PARP-1, all from Cell Signaling Technology (Denver, US), and tubulin [clone DM1A] (Sigma, St. Louis, USA). All immunoblots shown are representative of at least three independent experiments.

### Combination studies

In vitro combination studies were performed by seeding 96 well plates with SKLMS1 and STS39 cells (5000 c/well and 10000 c/well respectively). Cells were treated with BEZ235 and Dox at multiple concentrations: 4 × IC_50_, 2 × IC_50_, IC_50_, 0.5 × IC_50_, 0.25 × IC_50_ according to the following schedule: (1) concurrent treatment with Dox and selective inhibitor (SI) for 72 h, (2) SI alone for 24 h, followed by Dox for an additional 48 h and (3) Dox alone for 24 h followed by SI for an additional 48 h. Cell viability was quantified using the ATPlite assay on a PerkinElmer Enspire 2300 multimode plate reader and analyzed as described below.

### Treatment of LMS xenografts with BEZ235 and/or Dox

All in vivo work was carried out in accordance with the Animal Care Committee at the Toronto Centre for Phenogenomics, Toronto, Canada. Female NOD.Cg-*Prkdc*^*scid*^*IL2rg*^*tm1Wjl*^/SzJ mice were purchased from JAX Laboratories. At 6–8 weeks of age, animals received an intramuscular (i.m.) injection in the right hind limb with 5 × 10^6^ SKLMS1 cells. Once palpable, tumors were measured with callipers and tumor volumes calculated using the following formula: length × width × height × 0.5236 [24]. When tumors reached approximately 0.5 cm^3^ (~4–5 weeks for SKLMS1 cell lines), animals were assigned to four groups: Group 1. vehicle alone consisting of 10 % NMP (1-methyl-2-pyrrolidone)/PEG300 90 % daily by oral gavage and biweekly intraperitoneal (i.p) injection of PBS, Group 2. BEZ235 alone (25 mg/kg of BEZ235 daily by oral gavage dissolved in 10 % NMP/PEG300 90 % as described previously [25]), Group 3. Dox alone (1.2 mg/kg biweekly i.p. injection of Dox dissolved in PBS) and Group 4. concurrent treatment of BEZ235 and Dox. Body weight and tumor measurements were recorded twice a week for the duration of the study (12 days of treatment). Animals were euthanized by CO_2_ asphyxiation when tumors reached approximately 1.5 cm^3^ in size.

All tissues were stored either in liquid nitrogen or 10 % formalin. Xenograft tumors were stained for desmin (Dako), SMA [clone 1A4] (Dako), Ki-67 [clone SP6] (ThermoScientific), TUNEL (Roche, TdT) and p-AKT^S473^ (Cell Signaling Technology) at 1:100. Immunohistochemistry (IHC) was completed using standard protocols. For mouse primary antibodies the Mouse-on-Mouse Peroxidase Kit (Vector Laboratories, Burlington, ON, CAN) was used to prevent false positives to mouse-derived antigens.

### Statistics

Analysis of drug synergy was performed by calculating the combination index (CI) as a measure of interaction between two drugs. The CI was calculated according to the median- effect principle of the Chou and Talalay method using the CalcuSyn software 2.1 (BioSoft, UK) [26, 27]. Mann–Whitney test was performed to determine tumor volume differences between treatment groups. Differences of p < 0.05 were considered statistically significant. One-way ANOVA was performed to determine if there was any difference in mouse weight between treatment groups.

## Results

### Kinase inhibitor screen identifies PI3K and/or mTOR pathways as promising LMS targets

**Fig. 1:**
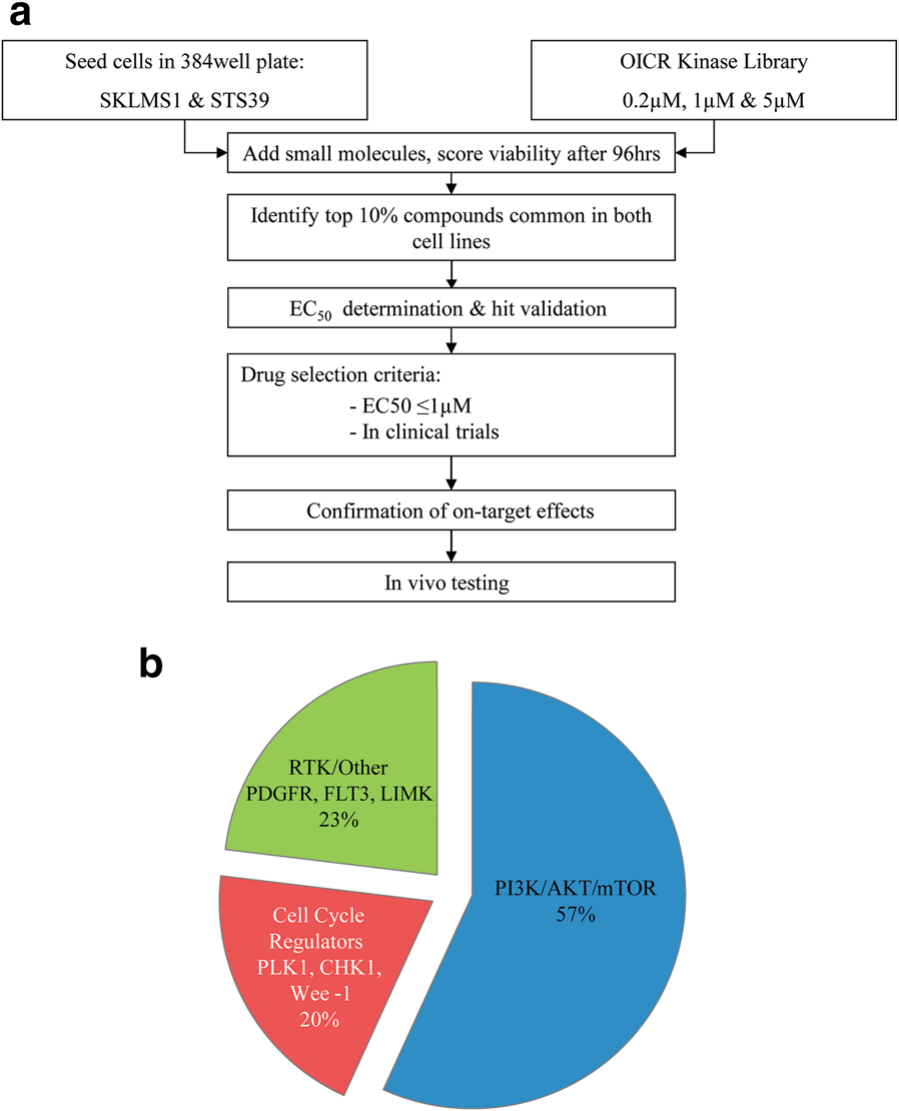
Primary screen with the OICR kinase library for possible novel therapies for LMS. **a** Flow chart detailing experimental procedure. **b** The top 10 % of hits from the primary screen is enriched for inhibitors targeting the PI3K/AKT/mTOR pathways. Other hits include cell cycle regulators, such as PLK1 and Wee-1, and RTK inhibitors, such as PDGFR

To identify effective therapies for LMS we screened two cell lines with a library of kinase inhibitors (Fig. 1a). Hits were defined as the top 10 % of compounds common across all dosages in both LMS cell lines (33 compounds). Hits were clustered on the basis of their primary kinase target (Fig. 1b). Interestingly, we identified a predominance of compounds that targeted the PI3K/AKT/mTOR pathways (52 %), cell cycle regulators (PLK1, CHK1, Wee-1) (27 %) and other RTK including PDGFR, and FLT3 (21 %). Overall, 20 (48 %) of the 42 PI3K/AKT/mTOR inhibitors included on the OICR plates were considered hits. We defined criteria for the selection of promising drugs as follows: hits that have nanomolar potency which predicts better clinical efficacy and/or compounds in phase I/II clinical trials at the time of our study design.

**Fig. 2:**
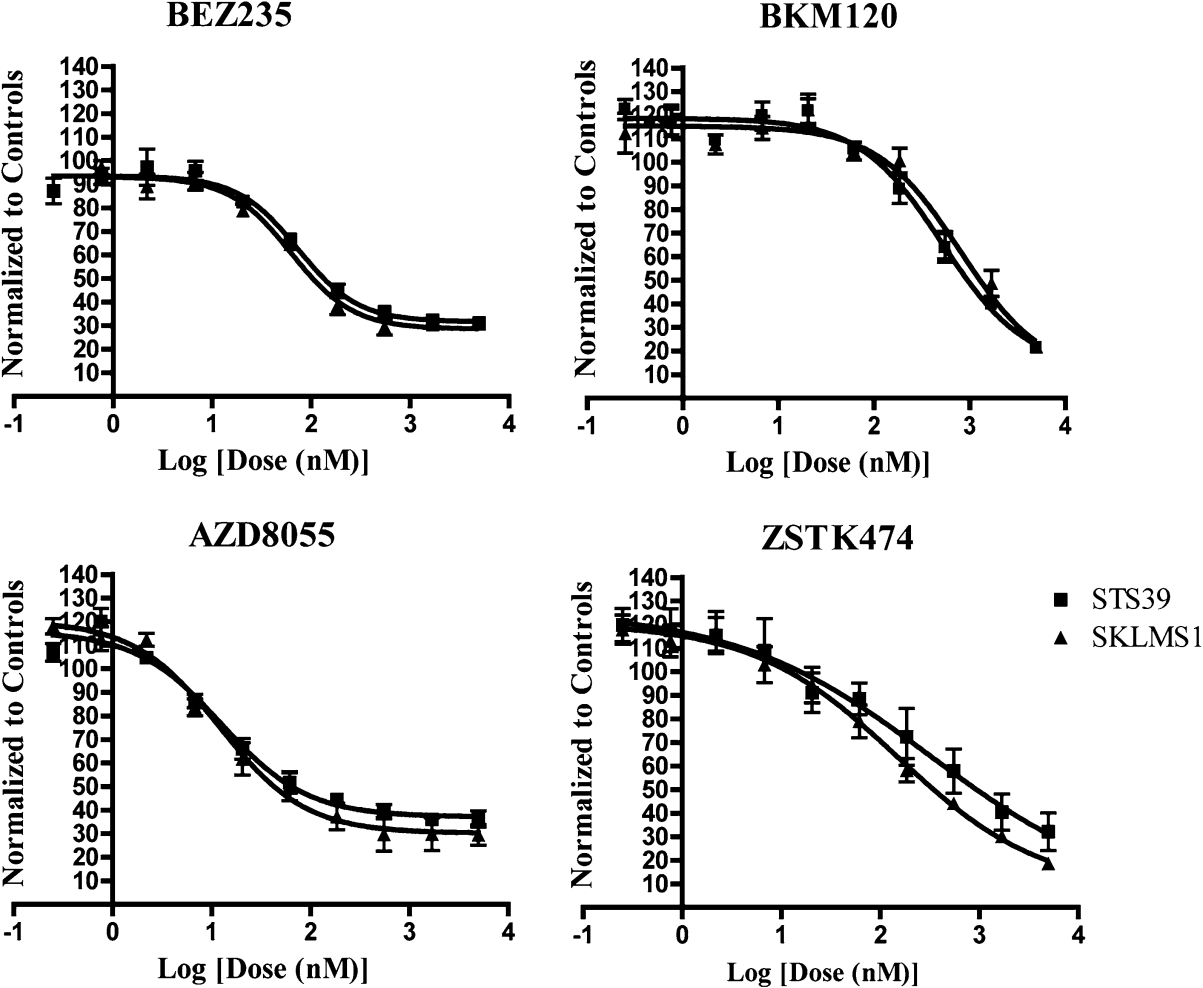
Secondary validation of selected hits discovered in primary screen in LMS cell lines. Treatment of SKLMS1 and STS39 cell lines with four inhibitors using a 10-point, threefold serial dilution, generating EC_50_ curves ranging from 0.25 to 5000 nM. Cells were incubated for 72 h and cell viability quantified with ATPlite (n = 3)

To validate selected hits from the primary screen, we retested 20 compounds and determined their EC_50_ values in both cell lines (Fig. 2). More than 70 % of primary hits were confirmed (14/20) in at least one cell line and 55 % of primary hits were confirmed in both cell lines (11/20). Furthermore, of 11 confirmed hits, 10 were molecules that targeted the PI3K and/or mTOR pathways, with the majority of the EC_50_ values less than 1 μM (Table 1). Of these, we investigated BEZ235 and BKM120 to determine their ability to inhibit LMS since both drugs are currently under investigation in Phase I/II trials and the low EC_50_ values indicate the possibility of a wide therapeutic window [28, 29]. BEZ235 is a dual ATP-competitive inhibitor for both PI3K and mTOR that demonstrated excellent nanomolar range potency (EC_50_ = 62.8 and 73.5 nM in SKLMS1 and STS39, respectively). BKM120 inhibits PI3K only and is somewhat less potent (EC_50_ = 800.0 and 513.2 nM in SKLMS1 and STS39, respectively).

**Table 1:** EC_50_ values generated for hits from primary screen

Pathway	Target	Drug	EC_50_ 72 h (nM)	
			SKLMS1	STS39
PI3K/AKT/mTOR	PI3K	PIK-75	10.79	14.9
		PIK-90	1705.3	952.2
		BKM120	800.0	513.2
		ZSTK474	149.0	318.8
		PF-04691502*	59.3	57.0
		BEZ235*	62.8	73.5
	AKT	A-443654	313.8	430.1
		GSK-690693	–	–
		MK-2206	933.5	486.9
	mTOR	AZD-8055	12.1	12.61
		OSI-027	–	–
		Everolimus	-	–
		KU0063794	434.3	240.1
		Rapamycin	–	–
Cell cycle regulators G2/M	Chk 1 and 2	AZD-7762	139.9	1001.0
	PLK1	BI-2536	1922.0	–
		GSK-461364	6.7	–
		BI-6727 (volasertib)	–	–
Cell cycle regulators G1/S	Wee-1	MK-1775	40.0	–
Other RTK	LIMK	LIM2K	–	–

EC_50_ values generated by the 20 hits found in the primary screen for both STS39 and SKLMS1. The 10-point dilution curve includes doses ranging from 0.25 to 5000 nM. Highlighted boxes are inhibitors used in further studies. Inhibitors with dash (–) indicate no EC_50_ value was generated, * indicates a dual inhibitor targeting both PI3K and mTOR

### Selective activity of BEZ235 and BKM120 in PI3K/mTOR pathways in LMS cell lines

We next analysed our cell lines to determine if they had any common mutations in the PI3K/mTOR axis. Since mutations in the p85 subunit of PI3K are a common mechanism of PI3K pathway activation in sarcomas [1], we sequenced both cell lines but did not identify activating mutations (data not shown). To determine if LMS cell lines were genomically stable with serial passaging, aCGH was performed which demonstrated that these cell lines were genomically stable over time (Additional file 1: Figure S1A). We also performed immunocytochemistry for desmin and SMA to confirm the primary cell line STS39 was representative of the original tumor it was derived from (Additional file 1: Figure S1B). Furthermore, we assessed endogenous PI3 K and mTOR pathway protein levels via immunoblot and found an increased level of p110 and p-p85 in SKLMS1 (Additional file 1: Figure S1C). RICTOR, a binding partner of mTOR whose activity has been implicated in carcinogenesis, was elevated in SKLMS1 consistent with a recent finding in well-differentiated LMS [30]. Finally, we demonstrated that generally protein levels of downstream effectors were consistently expressed with serial passaging (Additional file 1: Figure S1C).

**Fig. 3:**
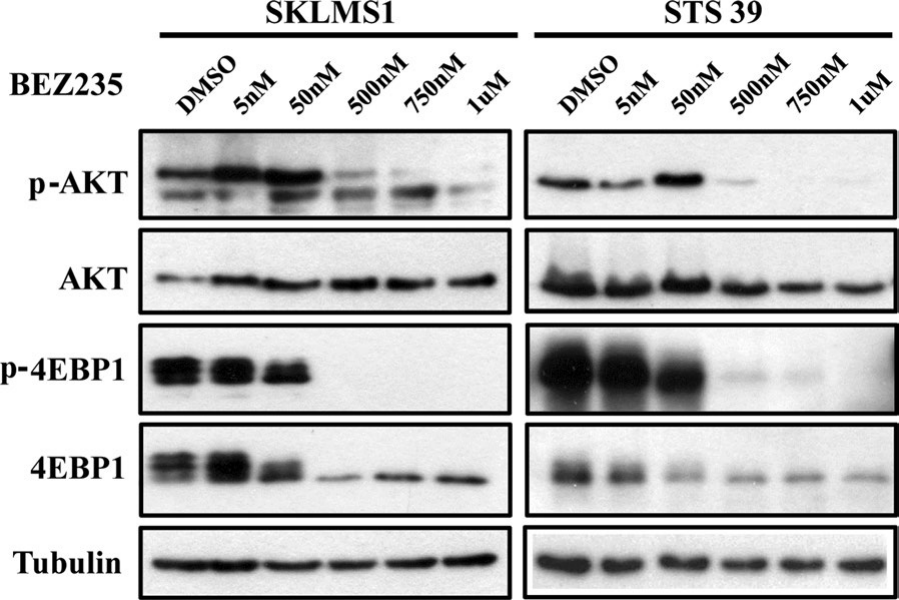
BEZ235 inhibits PI3K/mTOR pathway downstream effectors in LMS cells. Immunoblot demonstrates decreased levels of p-AKT^S473^ and of p-4EBP1^T37/46^ in total lysates from SKLMS1 and STS39 cells treated with BEZ235 for 72 h at concentrations ranging from 0 to 1000 nM/L. Total AKT, 4EBP1 and tubulin levels demonstrate equal loading of protein lysates

**Fig. 4:**
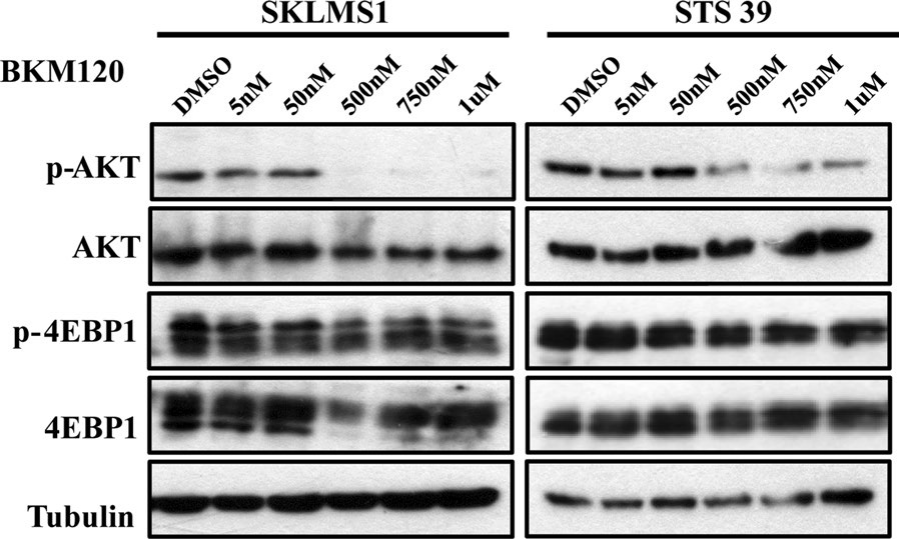
BKM120 inhibits PI3K but not mTOR pathway downstream effectors in LMS cells. Immunoblot demonstrating decreased levels of p-AKT^S473^, but not in p-4EBP1^T37/46^ in total lysates from SKLMS1 and STS39 cells treated with BKM120. SKLMS1 cells were treated for 72 h at concentrations ranging from 0 to 1000 nM/L. Total AKT, 4EBP1 and tubulin levels are shown for loading control

To determine if the PI3K/AKT/mTOR pathways are selectively inhibited in LMS cells, we treated both cell lines with BEZ235 or BKM120 for 72 h with five different doses (0–1000 nM). Dose–response experiments in STS39 and SKLMS1 cell lines demonstrated that BEZ235 inhibited the phosphorylation of AKT^S473^, a downstream target of PI3K. Inhibition of 4EBP1^T37/46^, a molecule downstream of the mTOR pathway was also observed at of 50 nM, however at higher concentrations (>500 nM) levels of 4EBP1 were decreased, in particular in SKLMS1 (Fig. 3). BKM120-treated cells demonstrated decreased levels of p-AKT^S473^ but not p-4EBP1^T37/46^ at a 500–1000 nM dose (Fig. 4). These data suggest that BEZ235 treatment in LMS cells inhibits downstream effectors of the PI3K and mTOR pathways and BKM120 treatment results in inhibition of PI3K pathway targets in both LMS cell lines as expected.

### Concurrent treatment with doxorubicin and BEZ235 is synergistic in LMS cell lines

Doxorubicin is the first line agent in adjuvant and metastatic settings in LMS patients [31]. Genetic aberrations in the PI3K/AKT pathway are becoming appreciated as common in STS and more recently in LMS [1, 32, 33]. Therefore, we tested the efficacy of Dox and PI3K and/or mTOR selective inhibitors to determine if combination therapy would result in synergy, which may allow for decreased drug doses thereby limiting toxicity and perhaps enhancing overall efficacy of drug therapy in LMS patients.

**Fig. 5:**
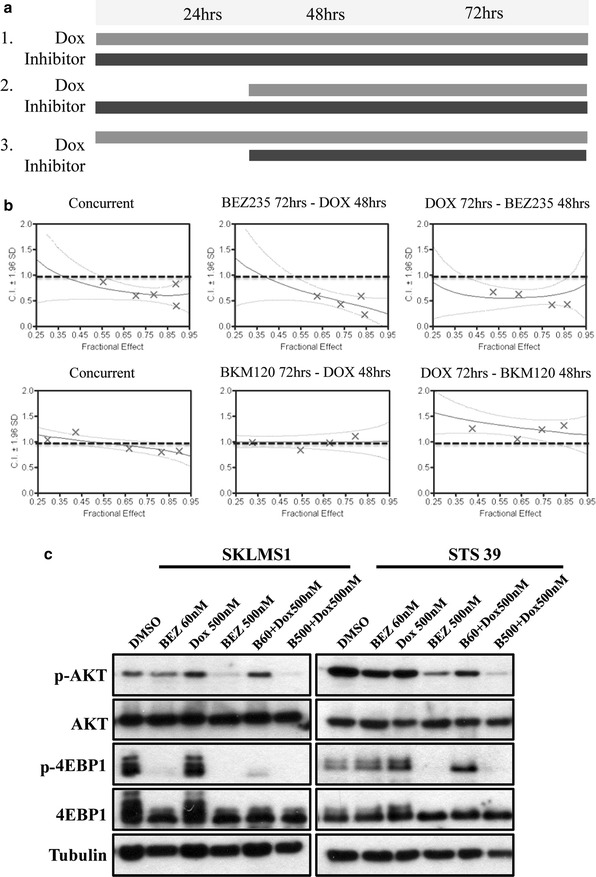
Combination studies of BEZ235 and Dox demonstrate synergy in LMS cell lines in vitro. **a** Dose schedule used in combination treatment. Three treatment schedules were investigated: schedule 1—concurrent treatment for 72 h, schedule 2—single agent therapy with inhibitor for the first 24 h followed by concurrent Dox treatment for the next 48 h and schedule 3—single agent Dox treatment for the first 24 h followed by concurrent treatment with the inhibitor for 48 h. **b** Combination index (CI) *graphs* resulting from the treatment of SKLMS1 cells with BEZ235 or BKM120 and Dox following 3 dosing schedules to determine optimal treatment regime. Viability was determined using ATPlite and analysed using CalcuSyn software. Treatment with BEZ235 (15–240 nM) and Dox (125–2000 nM) showed synergy in all 3 schedules (CI < 0.9), while combination BKM120 and Dox treatment was not synergistic in any of the treatment schedules (n = 3). For detailed CI ranges see Additional file 1: Table S2. **c** Immunoblot demonstrates decrease in p-AKT^S473^, and p-4EBP1^T37/46^ levels in total lysates from STS39 cells treated with BEZ235 for 72 h at the indicated concentrations and in combination with Dox. Total AKT, 4EBP1 and tubulin levels are shown as the loading controls

To determine an optimal therapeutic strategy we examined three dosing schedules: concomitant treatment for 72 h, or two different sequential treatments (inhibitor added 24 h after Dox treatment or Dox added 24 h after inhibitor) (Fig. 5a). In order to obtain an effective dosage range and dose density, the fixed (or constant) ratio for the two drug combination was calculated from the ratio of their EC_50_s data, as described by Chou-Talalay [27]. Specifically, BEZ235 and Dox were used at a fixed ratio of 1:4 to test for potential synergy and BKM120 and Dox were used together at a fixed ratio of 1:1. After the ratio was set, the mixture of the two drugs was serially diluted and the cells were incubated as per schedules described above. Cell viability was analysed with ATPlite.

Combination indexes (CI) were calculated for each dose combination. A CI < 1 indicated synergy, a CI = 1 indicated additive action and a CI > 1 indicated antagonism. Synergistic effects were observed with BEZ235 and Dox in both cell lines across a range of doses with median combination indices of 0.62 for SKLMS1 and STS39 (Fig. 5b). Synergy was observed during all three dosing schedules thus indicating that there is no difference between the treatment regimens. The combination of BKM120 and Dox resulted in an additive effects only (range of CI around 1.0) and thus was not pursued further in our in vivo studies (Additional file 1: Table S2).

We further investigated the capacity of BEZ235 and Dox to inhibit downstream effectors of PI3K/mTOR pathways, alone and in combination. Both LMS cell lines were treated either with BEZ235 at low (60 nM) or high (500 nM) concentrations with or without 500 nM Dox. BEZ235 (60 nM) alone and in combination with Dox was able to inhibit phosphorylation of 4EBP1^T37/46^ in SKLMS1 cells (Fig. 5c). Treatment of STS39 cells with BEZ235 at 60 nM caused a reduction in phosphorylation of AKT^S473^, with no change in the downstream effector p-4EBP1^T37/46^. As expected, increasing the dosage of BEZ235 to 500 nM dramatically decreased phosphorylation of AKT^S473^ and abolished the phosphorylation of 4EBP1^T37/46^. This effect was augmented with the addition of Dox. Thus, combination treatment causes decreased phosphorylation level of PI3K/mTOR downstream effectors in contrast to single Dox or BEZ235 treatment, reinforcing selective inhibition of these pathways, which warranted in vivo confirmation (Fig. 5c).

### BEZ235-Dox combination therapy induces cell death via apoptosis in vitro

To determine whether cell death following BEZ235 treatment was due to apoptosis, we evaluated Annexin V binding to the surface of drug-treated LMS cells with flow cytometry (Additional file 1: Figure S2). Annexin V-positive, 7-AAD-negative cells representative of early apoptotic cells and AnnexinV-positive, 7-AAD-positive cells indicative of late apoptotic cells were both present at low levels in cells treated with a single agent BEZ235 or Dox for 72 h (range 0.5–3.1 % of apoptotic cells). This cell population was increased with BEZ235 and Dox combination treatment for 72 h (15.3 and 9.6 % respectively for early and late apoptotic cells in SKLMS1, p < 0.01; 8 and 8.2 % respectively for early and late apoptotic cells in STS39, p < 0.01) suggesting that combination treatment may inhibit LMS survival via apoptosis. Furthermore, PARP-1 levels, a marker of apoptosis, were increased at concentrations of 500 nM BEZ235 and with BEZ235 and Dox combination treatment as compared to controls by immunoblot (data not shown).

### Concurrent treatment with doxorubicin and BEZ235 inhibits tumor growth in vivo

**Fig. 6:**
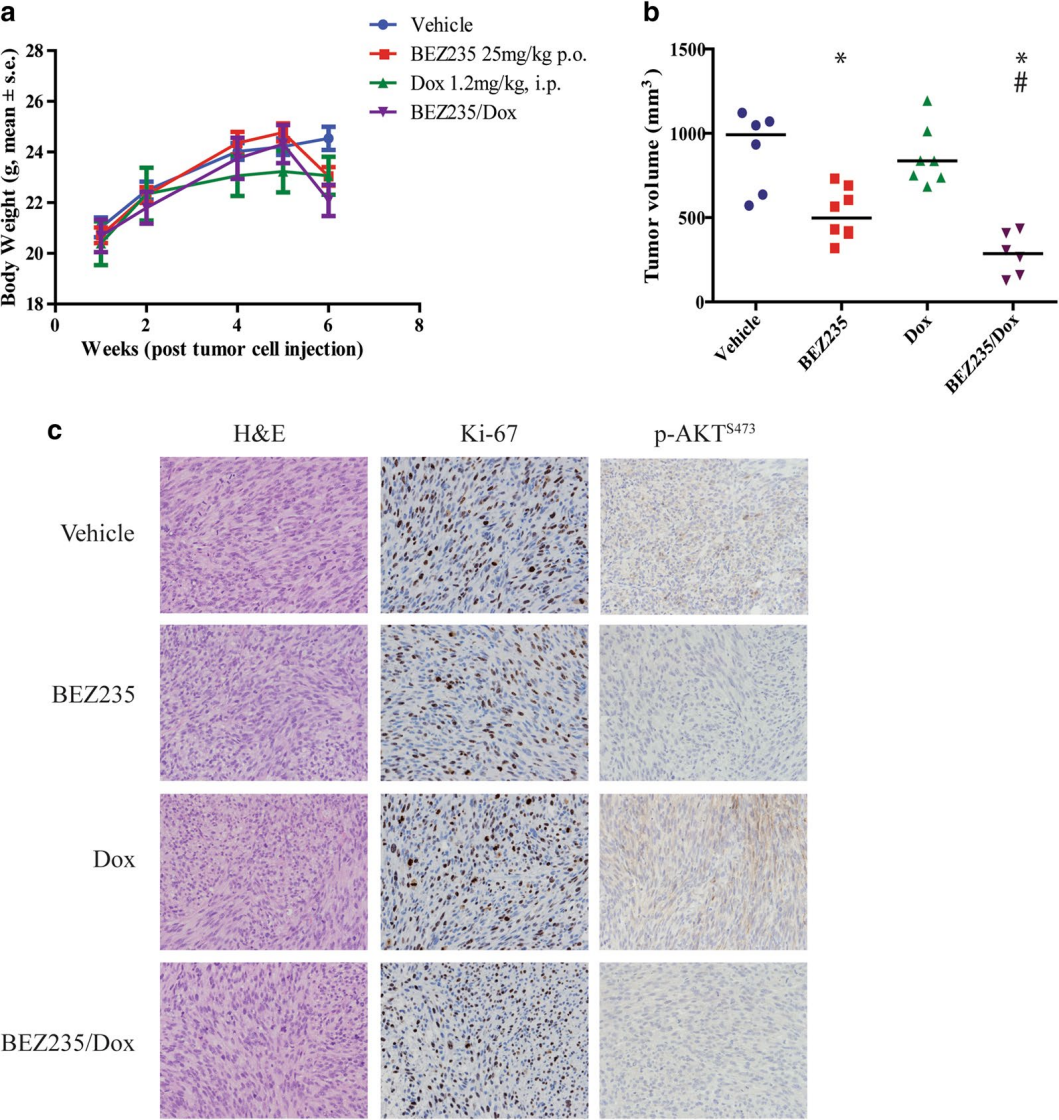
BEZ235/Dox combination inhibits LMS tumor growth in vivo. **a** Body weight is not significantly altered over the course of the experiment following administration of drugs in any treatment group (p = ns); **b** *Box plots* depict median tumor volume at experimental endpoint (after 12 days drug treatment), where vehicle (n = 6) had a median of 991.3 ± 236.0 mm^3^, while BEZ235 (n = 7), Dox (n = 7) and BEZ235/Dox (n = 6) groups had median volume of 498.3 ± 149.0, 836.6 ± 179.8, 286.7 ± 125.6 mm^3^ respectively (*p < 0.05 comparing treatment groups to vehicle, #p < 0.05 comparing BEZ235 treatment group with dual agent treatment group); **c** Representative photomicrographs demonstrating tumor morphology (H and E), cell proliferation (Ki-67), and p-AKT^S473^ status after treatment with BEZ235 or the combination of BEZ235 and Dox in SKLMS1 tumor xenograft specimens. All photomicrographs are 100× magnification

We next sought to determine if Dox and BEZ235 have synergistic effects in vivo. Thus, SKLMS1 cell lines were injected i.m. to recapitulate the microenvironment that is common in LMS formation. NSG mice bearing palpable tumors were randomized into 4 groups to receive vehicle, BEZ235, Dox, or a combination of both drugs. BEZ235 was administered for 12 days at 25 mg/kg po daily, which is a dose and schedule that have been shown to be efficacious for other in vivo cancer models [25]. Doxorubicin was given 1.2 mg/kg bi-weekly, i.p., which would achieve plasma levels comparable to human dosing [34, 35]. Treatment was well tolerated and body weight loss was <10 % of baseline in all treatment groups over the study period (Fig. 6a). Overall, tumor growth was inhibited by 50 % following administration of BEZ235 alone, which was further reduced by 71 % by combination therapy compared to vehicle only (Fig. 6b). Pathologically, both untreated and treated tumors were well demarcated, and comprised of spindle cells with eosinophilic cytoplasm and a fascicular pattern. The nuclei were ovoid and elongated with mild to moderate pleomorphism and brisk mitotic activity was seen in all cases (Fig. 6c). The tumour morphology and immunophenotype of the xenograft tumors was classified as LMS. No difference was observed in either Ki67 or TUNEL staining, markers of proliferation and apoptosis respectively, between the study groups (Fig. 6c and data not shown) in contrast to our in vitro data. Treatment with BEZ235 alone or in combination with Dox showed decreased in p-AKT^S473^ levels when compared with vehicle and Dox controls (Fig. 6c). Thus, dual treatment Dox and BEZ235 is more effective in inhibiting LMS tumor growth in vivo compared to single agent therapy. Although expression of proliferation and apoptosis were not statistically significant, in vivo regulation of AKT appeared to differ between treatment groups based on pAKT staining (Fig. 6c).

## Discussion

Advances in the development of selective therapeutic agents have resulted in exciting changes to the therapeutic landscape for solid tumors; however, success in treating sarcoma patients has remained limited. Activation of the PI3K/AKT/mTOR pathways through different mechanisms including activation of IGFR or PI3K, loss of PTEN, RICTOR amplification and/or increased p-AKT has been reported in LMS and other sarcoma subtypes [1, 36]. Recent clinical trials with selective PI3K and/or mTOR inhibitors have reported favourable efficacy and acceptable toxicity in solid tumors [37, 38].

### Kinase inhibitor screen identifies PI3K/AKT/mTOR inhibitors as potential therapeutic targets in LMS

In this pre-clinical study, we screened a collection of 480 kinase inhibitors using two LMS cell lines, one patient-derived and the other commercially available. Both cell lines were morphologically and immunophenotypically compatible with LMS and formed xenografts in vivo, thereby validating this LMS model system. Eleven potential hits were identified in our primary screen; with 10 out of 11 molecules targeting the PI3K and/or mTOR pathways (Table 1). Although several compounds matched the potency criteria, they were excluded from further analysis in this study because of poor performance in clinical trials. Therefore, two compounds that showed favourable selectivity profiles and were in clinical trials at the time of our study initiation, BEZ235 (a dual PI3K and mTOR inhibitor) and BKM120 (PI3K inhibitor) were chosen for further assessment. BEZ235 and BKM120 have shown efficacy in many types of cancer, such as breast cancer with activating PI3K mutations [39], ovarian cancer [40], pancreatic cancer [41], rhabdomyosarcoma [42, 43], hepatocellular carcinoma [44], undifferentiated pleomorphic sarcoma (UPS) in cell lines and/or animal models [45]. Although BEZ235 is no longer being marketed the field of development for PI3K/mTOR inhibitors (i.e. BYL719, PF-05212384; http://www.clinicaltrials.gov) is expanding clinically.

Dysregulated PI3K/AKT/mTOR signalling has been implicated in tumor progression and metastasis in multiple cancers of epithelial origin [46] and recent data has begun to elucidate that these signalling pathways may be critical in STS. Specifically, in a mouse model where PTEN, a known tumor suppressor, was inactivated using a conditional smooth muscle promoter, AKT activity played a critical role in smooth muscle transformation and LMS development [5]. Also zebrafish expressing constitutively active AKT^Ser473^ in mesenchymal progenitors resulted in the development of well-differentiated liposarcoma [32]. Furthermore, mutations in the PI3K receptor are frequently seen in myxoid round cell liposarcoma [1]. Finally, in a detailed pathologic assessment of human LMS RICTOR, a major component of the mTOR2 complex was significantly overexpressed [29].

### PI3K/mTOR pathway inhibition enhances doxorubicin-induced cell death in LMS

Doxorubicin is a potent anticancer drug used to treat several solid tumors including sarcoma. Despite being the primary treatment used for LMS, Dox has only partial efficacy, and its cardiotoxicity is a limiting factor. Furthermore, development of drug resistance to Dox is a major challenge undermining successful cancer treatment [10]. Dox has been shown to cause activation of AKT before apoptosis onset in cells [47]. This early induction of AKT may help confer chemotherapeutic resistance against Dox and provide a window in which to treat with PI3K/AKT inhibitors. Therefore, treatment of LMS by the combinations of PI3K/mTOR inhibitors with conventional chemotherapy agents may be beneficial, increasing the efficacy of Dox and thereby possibly improving the success of combination therapy in LMS patients.

Thus, we investigated whether combination of Dox and PI3K/mTOR inhibitors would have synergistic effects on LMS growth in vitro. Furthermore, we investigated whether these drug combinations should be administered concomitantly or sequentially by evaluating 3 treatment regimens: cells pre-treated with either drug, or concurrent treatment. CI results indicated that the anti-proliferative effect of the combination of BEZ235 and Dox was synergistic in both LMS cell lines at all dose levels tested. However, the treatment of LMS cells with the combination of BKM120 and Dox did not result in synergy. Based upon these results, in vivo experiments were performed using only the BEZ235 and Dox combination in an intra-muscular xenograft model of LMS. Although single agent Dox did not significantly inhibit tumor growth compared to control animals, administration of BEZ235 alone or in combination with Dox resulted in significant reduction in tumor volume. Our findings are similar to reports from Kirsch et al. who showed a response rate of 11.1 % after BEZ235 treatment which increased to 50 % upon combination with Dox when treating another common sarcoma subtype UPS [45]. I.

## Conclusions

In summary, we demonstrate that inhibition of the PI3K and mTOR pathways impairs LMS growth in vitro and in vivo. Also, we describe that the use of PI3K and mTOR inhibitors may have a synergistic effect with doxorubicin, standard chemotherapy for this disease. Thus, future studies with inhibitors targeting these pathways are warranted.

## Additional file

10.1186/s12967-016-0814-z Characterisation of LMS cell lines: A. Heat map of copy number variations between STS39 tumor, STS39 cell lines passages 4, 9, 14, 24, 34, SKLMS1 and HUVEC (Human umbilical vein endothelial cells) as a control, showing genomic stability of cell lines over time. The scale represents the percentage of genetic differences, where white represents minimal to no genetic change and dark purple represents maximum genetic change. For example, the genetic difference between HUVEC and STS39 is 23%. B. Immunocytochemistry of both cell lines using DAPI, Desmin, Smooth Muscle Actin (SMA) and mouse IgG (msIgG) as an isotype control. SKLMS1 demonstrates focally positive SMA staining, while STS39 shows focal positivity for Desmin and SMA (n=3). C. Immunoblot analysis showing protein stability of PI3K pathway proteins with serial passaging of STS39 and SKLMS1 cells. Increased phosphorylation of p85, a subunit of the PI3K receptor, was seen in SKLMS1 cells. In addition, elevated levels of RICTOR, a binding partner of mTOR, were observed. Both of these modifications can potentially lead to increased pathway activation. HeLa and Jurkat cell lines were used as controls for protein expression. siRNA against 4EBP1 was used to create a negative control for 4EBP1 and p-4EBP1 antibodies. **Figure S2.** Treatment with BEZ235 and/or Dox induces cell death via apoptosis. A. Cells were treated with BEZ235 (500nM), Dox (500nM) and BEZ/Dox for 72h and then analysed for apoptosis by flow cytometry for Annexin V and 7-ADD staining. Combination of BEZ235 and Dox significantly induced apoptosis in SKLMS1 and STS-39 cells (data not shown). B. Quantification of apoptotic cells at 72h post-treatment of SKLMS1 cells (n=3) and STS39 cells (n=3). **Table S1.** Sequencing primers (5′-3′) used to determine the presence of mutations in mTOR and in exon 9 and 20 of the kinase domain of PI3K. **Table S2.** Combination Index (CI) tables with BEZ235, BKM120 and/or Dox at 3 dosing schedules. Viability was determined using an ATPlite assay and analysed using CalcuSyn software. Treatment with BEZ235 (15-240nM) and Dox (125-2000nM) showed synergy in all 3 schedules (CI<0.9), while the combination of BKM120 and Dox was not synergistic (n=3).

## Abbreviations

LMS: leiomyosarcoma
Dox: doxorubicin
STS: soft tissue sarcomas
RTK: receptor tyrosine kinases
aCGH: array comparative genomic hybridization
STR: short tandem repeat
TCAG: The Centre for Applied Genomics
OICR: Ontario Institute for Cancer Research
LTRI: Lunenfeld-Tanenbaum Research Institute
DMSO: dimethylsulfoxide
SI: selective inhibitor
NMP: 1-methyl-2-pyrrolidone
CI: combination index
