# Supplementary material for: PI3K/AKT/mTOR inhibition in combination with doxorubicin is an effective therapy for leiomyosarcoma
Source: J Transl Med. 2016 Mar 8;14:67. doi: 10.1186/s12967-016-0814-z (PMC4782390; doi:10.1186/s12967-016-0814-z)
Supplement: Supplementary file 1 — 10.1186/s12967-016-0814-z Characterisation of LMS cell lines: A. Heat map of copy number variations between STS39 tumor, STS39 cell lines passages 4, 9, 14, 24, 34, SKLMS1 and HUVEC (Human umbilical vein endothelial cells) as a control, showing genomic stability of cell lines over time. The scale represents the percentage of genetic differences, where white represents minimal to no genetic change and dark purple represents maximum genetic change. For example, the genetic difference between HUVEC and STS39 is 23%. B. Immunocytochemistry of both cell lines using DAPI, Desmin, Smooth Muscle Actin (SMA) and mouse IgG (msIgG) as an isotype control. SKLMS1 demonstrates focally positive SMA staining, while STS39 shows focal positivity for Desmin and SMA (n=3). C. Immunoblot analysis showing protein stability of PI3K pathway proteins with serial passaging of STS39 and SKLMS1 cells. Increased phosphorylation of p85, a subunit of the PI3K receptor, was seen in SKLMS1 cells. In addition, elevated levels of RICTOR, a binding partner of mTOR, were observed. Both of these modifications can potentially lead to increased pathway activation. HeLa and Jurkat cell lines were used as controls for protein expression. siRNA against 4EBP1 was used to create a negative control for 4EBP1 and p-4EBP1 antibodies. Figure S2. Treatment with BEZ235 and/or Dox induces cell death via apoptosis. A. Cells were treated with BEZ235 (500nM), Dox (500nM) and BEZ/Dox for 72h and then analysed for apoptosis by flow cytometry for Annexin V and 7-ADD staining. Combination of BEZ235 and Dox significantly induced apoptosis in SKLMS1 and STS-39 cells (data not shown). B. Quantification of apoptotic cells at 72h post-treatment of SKLMS1 cells (n=3) and STS39 cells (n=3). Table S1. Sequencing primers (5′-3′) used to determine the presence of mutations in mTOR and in exon 9 and 20 of the kinase domain of PI3K. Table S2. Combination Index (CI) tables with BEZ235, BKM120 and/or Dox at 3 dosing schedules. Viability [file 12967_2016_814_MOESM1_ESM.pdf]

## SUPPLEMENTAL METHODS

### aCGH Analysis

STS39 and SKLMS1 cell lines were analysed for copy number variation using array comparative genomic hybridization on the Genome-Wide Human SNP Array 6.0, and mapped using STR analysis (TCAG, Sick Kids, Toronto, CA). aCGH analysis was performed using the oligo package (v.1.12.2), in the R statistical environment (v.2.11.1), and data visualization performed using the lattice (v.0.20-24), and latticeExtra (v.0.6-26) packages in the R statistical environment (v.3.0.2).

### Annexin V assay

The ability of BEZ235 or Dox to induce apoptosis in LMS cell lines was determined with an Annexin V-PE detection kit, used according to the manufacturer's instructions (Affymetrix eBioscience, San Diego, USA). Briefly, SKLMS1 and STS39 cells were treated with BEZ235 (500nM) and/or Dox (500nM) for72h, cells were then harvested and stained with Annexin V-PE and 7-Aminoactinomycin D (7-AAD) Viability Staining Solution. Analysis was performed on a LSR II flow cytometer (BD). Relative numbers of Annexin V–positive and 7-AAD-positive or negative cells were obtained for each cell line and analyzed using FlowJo vX.

**ADDITIONAL FILE LEGENDS:**

**Additional file 1: Figure S1.** Characterisation of LMS cell lines: A. Heat map of copy number variations between STS39 tumor, STS39 cell lines passages 4, 9, 14, 24, 34, SKLMS1 and HUVEC (Human umbilical vein endothelial cells) as a control, showing genomic stability of cell lines over time. The scale represents the percentage of genetic differences, where white represents minimal to no genetic change and dark purple represents maximum genetic change. For example, the genetic difference between HUVEC and STS39 is 23%. B. Immunocytochemistry of both cell lines using DAPI, Desmin, Smooth Muscle Actin (SMA) and mouse IgG (msIgG) as an isotype control. SKLMS1 demonstrates focally positive SMA staining, while STS39 shows focal positivity for Desmin and SMA (n=3). C. Immunoblot analysis showing protein stability of PI3K pathway proteins with serial passaging of STS39 and SKLMS1 cells. Increased phosphorylation of p85, a subunit of the PI3K receptor, was seen in SKLMS1 cells. In addition, elevated levels of RICTOR, a binding partner of mTOR, were observed. Both of these modifications can potentially lead to increased pathway activation. HeLa and Jurkat cell lines were used as controls for protein expression. siRNA against 4EBP1 was used to create a negative control for 4EBP1 and p-4EBP1 antibodies.

**Figure S2.** Treatment with BEZ235 and/or Dox induces cell death via apoptosis.
A. Cells were treated with BEZ235 (500nM), Dox (500nM) and BEZ/Dox for 72h and then analysed for apoptosis by flow cytometry for Annexin V and 7-ADD staining. Combination of BEZ235 and Dox significantly induced apoptosis in SKLMS1 and STS-39 cells (data not shown). B. Quantification of apoptotic cells at 72h post-treatment of SKLMS1 cells (n=3) and STS39 cells (n=3).

**Table S1.** Sequencing primers (5′-3′) used to determine the presence of mutations in mTOR and in exon 9 and 20 of the kinase domain of PI3K.

**Table S2.** Combination Index (CI) tables with BEZ235, BKM120 and/or Dox at 3 dosing schedules. Viability was determined using an ATPlite assay and analysed using CalcuSyn

software. Treatment with BEZ235 (15-240nM) and Dox (125-2000nM) showed synergy in all 3 schedules (CI<0.9), while the combination of BKM120 and Dox was not synergistic (n=3).

## SUPPLEMENTAL FIGURES AND TABLES

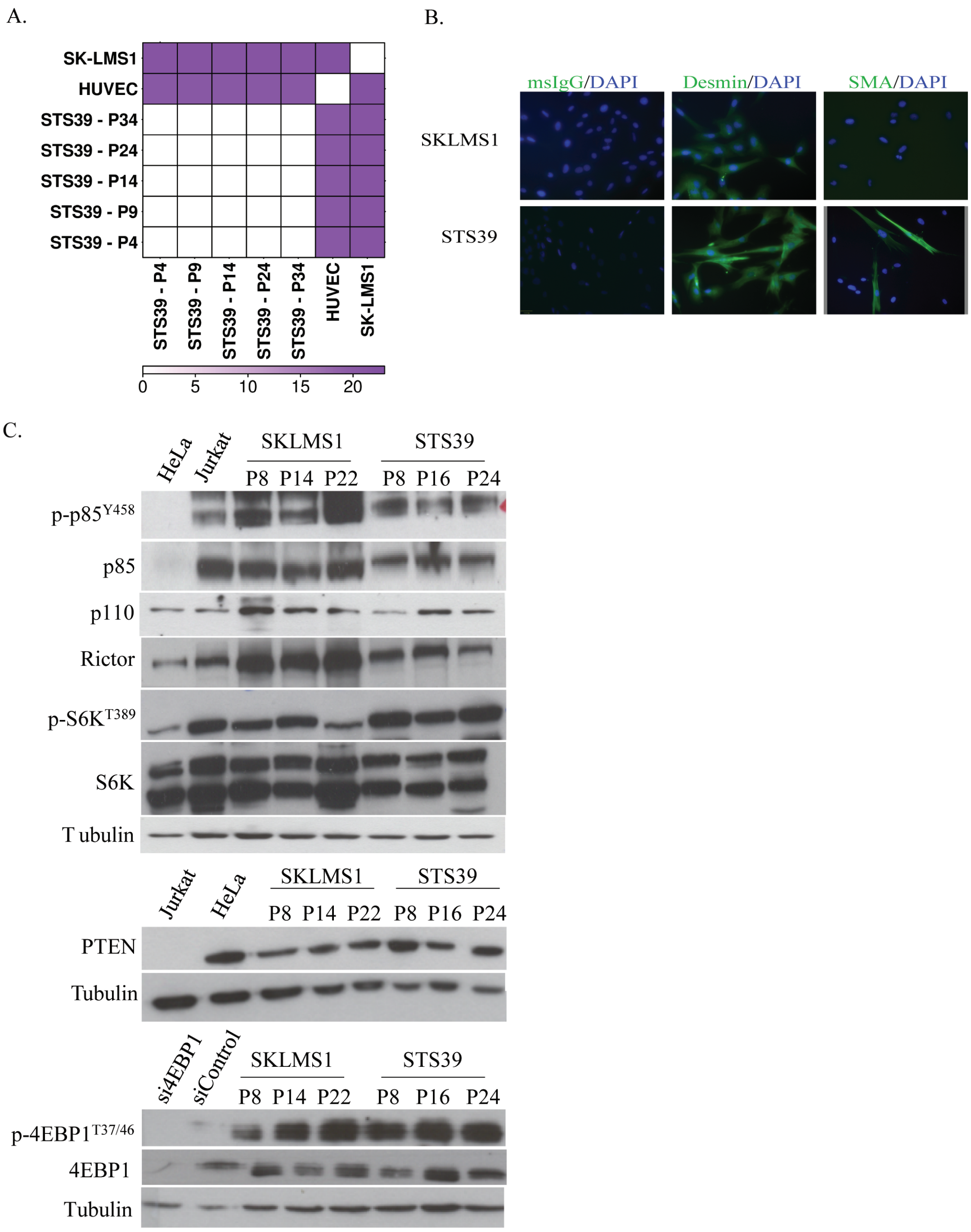


**Supplemental Figure 1**

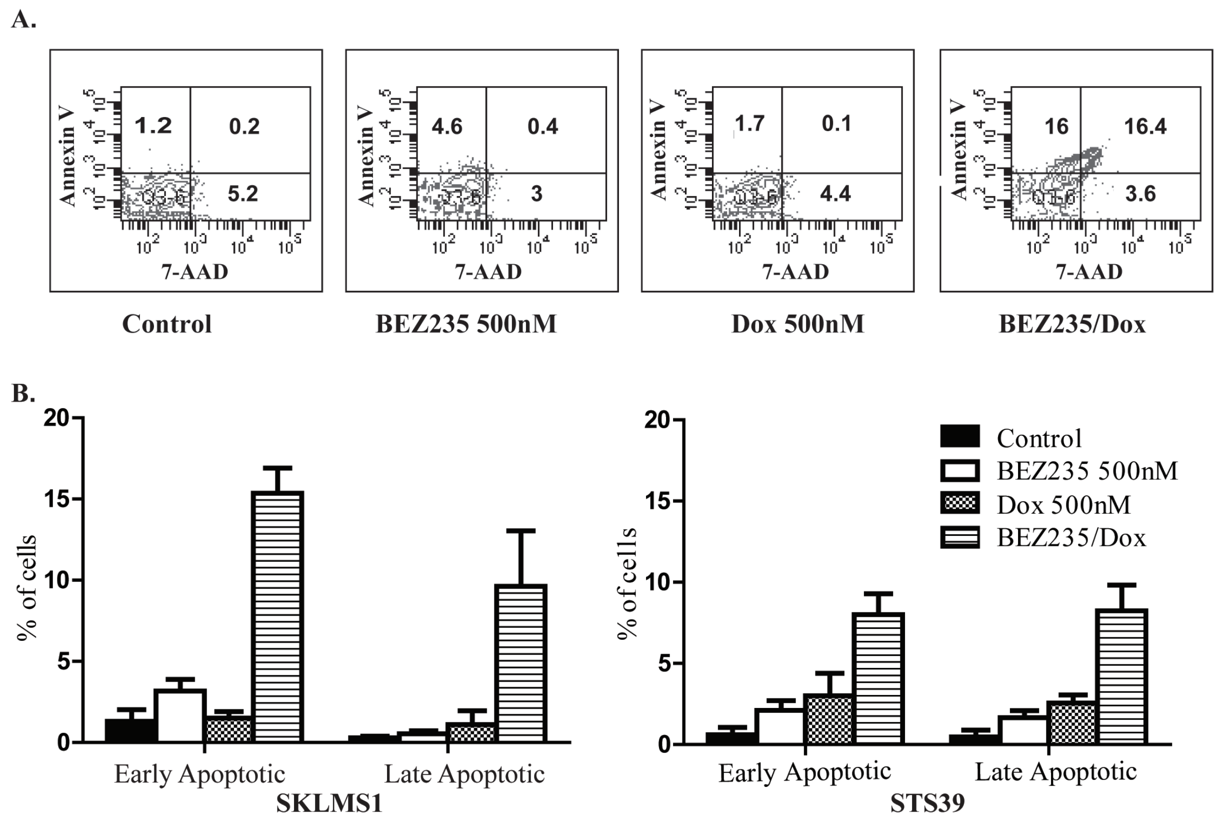


**Supplemental Figure 2**

| Gene Name | Sequencing Primer |
|---|---|
| mTOR (exon 14-15) | CCTCATTTCCAGGCCACTAA |
| PI3K exon 9 (F) | TTGCTTTTTCTGTAAATCATCTGTG |
| PI3K exon 9(R) | TGACATTTGAGCAAAGACCTG |
| PI3K exon 9 (S) | TTGCTTTTTCTGTAAATCATCTGTG |
| PI3K exon 20(F) | TGGGGTAAAGGGAATCAAAAG |
| PI3K exon 20(R) | CCTATGCAATCGGTCTTTGC |
| PI3K exon 20(S) | TGACATTTGAGCAAAGACCTG |
| PI3K exon 20(F) | TTGCATACATTCGAAAGACC |
| PI3K exon 20(R) | GGGGATTTTTGTTTTGTTTTG |
| PI3K exon 20(S) | TTTGTTTTGTTTTGTTTTTT |

**Supplemental Table 1**

| SKLMS1 drug dose | | CI For experimental values | | | CI For experimental values | | |
|---|---|---|---|---|---|---|---|
| Dox(nm) | Bez(nm) | Dox/Bez 72h | Bez 72h/Dox 48h | Dox 72h/BEZ 48h | Dox/BKM 72h | BKM 72h/Dox 48h | Dox 72h/BKM 48h |
| 125 | 15 | 0.871 | 0.977 | 0.676 | 1.046 | 1.122 | 1.971 |
| 250 | 30 | 0.601 | 0.594 | 0.632 | 1.192 | 0.987 | 1.258 |
| 500 | 60 | 0.62 | 0.436 | 0.424 | 0.869 | 0.837 | 1.051 |
| 1000 | 120 | 0.399 | 0.235 | 0.428 | 0.8 | 0.983 | 1.237 |
| 2000 | 240 | 0.835 | 0.591 | 1.041 | 0.817 | 1.116 | 1.325 |

| STS39 drug dose | | CI For experimental values | | | CI For experimental values | | |
|---|---|---|---|---|---|---|---|
| Dox(nm) | Bez(nm) | Dox/Bez 72h | Bez 72h/Dox 48h | Dox 72h/BEZ 48h | Dox/BKM 72h | BKM 72h/Dox 48h | Dox 72h/BKM 48h |
| 125 | 15 | 0.598 | 0.997 | 0.789 | 1.155 | 0.982 | 0.807 |
| 250 | 30 | 0.563 | 0.885 | 0.635 | 2.642 | 1.068 | 1.11 |
| 500 | 60 | 0.62 | 0.688 | 0.643 | 1.418 | 0.902 | 1.002 |
| 1000 | 120 | 0.68 | 0.838 | 0.839 | 1.504 | 0.997 | 1.127 |
| 2000 | 240 | 0.376 | 1.123 | 0.537 | 1.046 | 0.872 | 1.13 |

**Supplemental Table 2**
